# DArTSeq SNP-based genetic diversity and population structure studies among taro [(*Colocasia esculenta* (L.) Schott] accessions sourced from Nigeria and Vanuatu

**DOI:** 10.1371/journal.pone.0269302

**Published:** 2022-11-10

**Authors:** Tilahun Wondimu Fufa, Wosene Gebreselassie Abtew, Charles Okechukwu Amadi, Happiness Ogba Oselebe

**Affiliations:** 1 Department of Horticulture, Oromia Agricultural Research Institute, Addis Ababa, Ethiopia; 2 Department of Crop Production and Landscape Management, University of Ebonyi State, Abakaliki, Nigeria; 3 Department of Horticulture and Plant Science, Jimma University, Jimma, Ethiopia; 4 Department of Cocoyam Improvement, National Root Crops Research Institute, Umudike, Nigeria; National Agri-Food Biotechnology Institute (NABI) Mohali, INDIA

## Abstract

Taro is a valuable staple food crop among resource-poor rural people in countries such as Nigeria and Ghana, among others. Characterization of genetic diversity is a prerequisite for proper management of breeding programs and conservation of genetic resources. Two hundred seventy one taro accessions obtained from Nigeria and Vanuatu were genotyped using DArTseq-based SNP markers with the objectives of investigating the genetic diversity and population structure. In the analysis, 10,391 SNP markers were filtered from the sequence and used. The analysis revealed higher transition than transversion types of SNPs in the ratio of 1.43:1. The polymorphism ranged from 0.26 to 0.29 for the markers, indicating moderate genetic diversity. A model-based Bayesian clustering analysis of taro accessions yielded five subgroups and revealed the admixture situation in 19.19% of all accessions in the study. Vanuatu taro accessions exhibited more genetic diversity than Nigerian taro accessions. The population diversity estimate (PhiPt) was relatively higher (0.52) for accessions originating from Vanuatu than for Nigerian accessions. Analysis of molecular variance (AMOVA) revealed that most variation existed among individuals within a population at 52%. Nei’s genetic distance showed that relatedness is based on geographical proximity. Collection of taro genetic resources should give more emphasis to within regions to utilize diversity in taro breeding program. This study also demonstrated the efficiency of DArTseq-based SNP genotyping for large-scale genome analysis in taro. The genotypic markers provided in this study are useful for association mapping studies.

## Introduction

Taro is one of the oldest food crops, dating back over 9,000 years and has history of 2000 years in cultivation [1]. High diversity of taro was reported in south East Asia while its origin was reported to be South Central Asia [2]. Taro has continued to spread throughout the world and is now an important crop in Asia, the Pacific, Africa and Caribbean. According to FAOSTAT record of 2018, about 10.64 million tons of taro were produced globally from 1.66 million hectares with an average yield of 9.5 t ha^-1^ [3]. The same year Nigeria, the largest taro producer worldwide, harvested about 3.33 million tons from 0.72 million hectares with the average yield of 4.14 t ha^-1^.

Taro is staple food [4] and regularly consumed as a main component or as soup thickener in the south eastern parts of Nigeria [5]. Primarily taro is grown for its starchy corm [6] and rarely leaves, petioles and inflorescences are also edible [1]. It contains substantial amounts of minerals and vitamins with lesser amounts of fats, fibers, and ash. It can aid diabetic patients, the aged people, and children with allergy and for other persons with intestinal disorders [7].

The biosphere has more than 30,000 plant species that are thought to be edible [8]. Taro is ranked 19^th^ among the world’s top 20 edible food crops [9]. Between 1970 and 1980 taro was among the three most consumed staple food crops in Nigeria [5]. Despite its growing importance as a crop in many parts of the world and its cultural significance among users, no international agricultural research center has a mission to conserve and do research on taro [10]. Even though some efforts have been made in Philippine, Fiji, Papua New Guinea and other countries in Oceania [11], there is no Nigerian germplasm repository responsible for conserving taro [12]. For many years, taro has been maintained by farmers and its genetic resources have remained largely under the control of local communities. It is produced by small scale farmers [4] and its commercial importance is also largely local. This implies that farmers have been the main users and custodians of taro genetic diversity with constant selection for their traits of preference. Thus, the exploitation of this diversity could lead to the development of cultivars with greater disease resistance, improved yields and corm quality.

Many researchers had reported on genetic variation among taro accessions. High genetic diversity were reported in Asian taro accessions using AFLP markers [13], simple sequence repeat (SSR) [2], RAPD [14], isozymic patterns markers [15] and microsatellite markers [16, 17]. Multiple ploidy was reported from mainland Asia and more diploids were found in the Pacific Islands [18]. West African taro were reported for having linage with the diploid Asian taro accessions [2].

In terms of recent advances in molecular markers such as SSR and single nucleotide polymorphism (SNP), taro is an orphan crop. SSR and SNP are the two most reliable markers for assessing genetic diversity and population structure in any organism. SNP markers have a higher population resolution than SSR markers [19]. In this regard, none of the first generation molecular markers used to assess genetic diversity among Nigerian taro accessions were found to be effective. Efforts to preserve the original Nigerian taro accessions will benefit from the use of DNA-based methods such as SNPs for genetic stock identification and the use of useful genes in taro breeding programs. Moreover, genome-wide exploration of genetic relatedness and diversity of Nigerian taro is still missing.

Taro is an important crop in the Asia-Pacific region’s agriculture [20]. It is especially important in Oceania, and no other region of the world can match Oceania in terms of the intensity of production, utilization, and reliance on taro for food. Most Oceania cultures have evolved on the strength of root crops as the primary food source, and taro is still one of the top two or three staple foods in the majority of them today. Small-scale farmers in Vanuatu (Oceania) still rely heavily on the sustainable use and maintenance of a diverse biodiversity, with root and tuber crops providing the majority of daily subsistence [21]. Taro is a staple crop in Vanuatu [22] and the national *ex-situ* collection in Vanuatu contains 125 taro varieties from most of the islands [23]. The Nigerian cocoyam research department recently introduced some taro accessions to test their adaptability in Nigerian conditions. This set includes 94 hybrid seeds that were introduced. Thus, this study was aimed to investigate the genetic diversity and population structure among taro accessions sourced from Nigerian and Vanuatu regions. Moreover, the scope of differentiation was evaluated. As a result, we contribute to taro germplasm conservation and breeding initiatives.

## Materials and methods

### Plant materials

Two hundred eighty two taro accessions were used in this study, of which 94 accessions were collected from different regions of Nigeria including Enugu, Ebonyi, Imo, Anambara and Abia. The 188 taro accessions were kindly provided by National Root Crop Research Institute of Nigeria (NRCRI) of which 94 accessions were imported from Vanuatu and the remaining 94 taro accessions were obtained from NRCRI in situ conservation. All materials obtained from Vanuatu were hybrids whereas materials from Nigeria were landraces. The S1 Table contains the details of the accessions used in the study.

### DNA extraction and sequencing

Two hundred eighty two taro accessions were grown at the University of Ebony state (Nigeria) teaching and experimental nursery on 28^th^ May 2019. Taro leaves were sent to Integrated Genotyping Service and Support (IGSS) platform, currently SEQART AFRICA located at Biosciences Eastern and Central Africa (BecA-ILRI) Hub in Nairobi for Genotyping. DNA extraction was done using Nucleomag Plant DNA extraction kit. The genomic DNA extracted was in the range of 50-100ng/ul. DNA quality and quantity were checked on 0.8% agarose. Libraries were constructed according to Kilian et al. [24]. DArTSeq complexity reduction method was used through digestion of genomic DNA with two restriction enzymes (*PstI* and *MseI*) and ligation of barcoded adapters followed by PCR amplification of adapter-ligated fragments. Libraries were sequenced using single read sequencing runs for 77 bases by Hiseq2500. DArTseq markers scoring was achieved using DArTsoft14 which is an in-house marker scoring pipeline based on algorithms. Two types of DArTseq markers were scored, SilicoDArT markers and SNP markers which were both scored as binary for presence /absence (1 and 0, respectively) of the restriction fragment with the marker sequence in genomic representation of the sample. Both SilicoDArT markers and SNP markers were aligned to the reference genomes of Taro (Taro_V1), to identify chromosome positions.

### Genetic diversity

For quality control, DArTseq SNP markers were filtered to remove unwanted SNP markers using the software PLINK 1.9 and VCFtools [25]. Markers and genotypes with a missing data rate greater than 25% were removed. Rare SNPs with minor allele frequencies of 5% were also removed. Only 10, 391 DArT-SNP markers and 271 cultivars were found to be useful in the subsequent analysis. To estimate marker statistics such as minor allele frequency (MAF), observed heterozygozity (H_o_), expected heterozygozity (H_e_), and polymorphic information (PIC) content, the R package "adegenet" [26] was used. To determine mutation transversion (TV) and transition (TS), the SniPlay web [27] base was used. Plink’s recodeA function was used to generate the dosage SNP format 0, 1, 2, where 0 represents the homozygote reference, 1 represents the heterozygote, and 2 represents the homozygote alternative. The GenAIEx ver. 6.5 software [28] was used to perform AMOVA to divide the total level of genotypic variance into variance within and between populations and sources of collection. The Mantel test was used to compare the genetic distance [29] and geographic distance matrices between populations. AMOVA was used to calculate the genetic differentiation between the PhiPT populations (analog of F_ST_). 999 permutations were used to determine the statistical significance of the AMOVA and Mantel tests for all populations and loci [30].

### Population structure

The binary file generated from the VCF file was subjected to admixture analysis with the “adegenet” R package [31]. Using k-means analysis, the optimal number of clusters was determined after varying the number of clusters from 2 to 100 and various clustering solutions were compared using the Bayesian Information Criterion (BIC) [32]. The number of clusters corresponding to the lowest BIC, i.e., an elbow in the curve of BIC values as a function of k, was determined. Using the admixture analysis, genotypes with membership proportions (Q-value) greater than or equal to 60% was assigned to groups. Those with membership probabilities of less than 60% were labeled as admixtures [33].

## Results

### SNP summary

After preprocessing and filtering, 271 taro accessions and 10,391 SNP markers were retained. These 10,391 SNP markers were mapped onto 14 taro chromosomes, with an average of 742 SNP markers per chromosome (S2 Table). In total, more TS type SNPs (59%) than TV type SNPs (41%) were found in the genomes of the taro accessions studied (Table 1).

**Table 1:** SNP Mutations of transition and transversion types.

Mutation	Transition (TS)		Transversion (TV)			
SNP type	AG	CT	AC	AT	CG	GT
Quantity	3102	3018	1071	1234	907	1059

The genetic parameter estimates, i.e. H_o_, H_e_, MAF, and PIC of the 10,391 SNP markers from 271 taro accessions are presented in (Table 2). The average H_o_ in this study was 0.47, while H_e_, MAF, and PIC were 0.33, 0.29, and 0.25, respectively. The hybrids (Vanuatu accessions) showed relatively higher genetic diversity than landraces (Nigerian accessions) (Table 2).

**Table 2:** A summary of marker statistics.

Population	H_o_	H_e_	MAF	PIC
Landrace (Nigeria)	0.40	0.26	0.23	0.20
Hybrid (Vanuatu)	0.53	0.39	0.35	0.30
Average	0.47	0.33	0.29	0.25

### Genetic diversity and population structure

#### Analysis of molecular variance (AMOVA)

Table 3 shows the results of AMOVA for the 271 taro accessions using 10,391 SNP markers. The results showed a high (47%) and highly significant variation among regions. Individuals within the population showed high (52%) and highly significant variation. However, the variance among populations is low (1%) and non-significant.

**Table 3:** Analysis of molecular variance (AMOVA) results for PhiRT, PhiPR and PhiPT statistics probability, (rand > = data), based on standard permutation (999) across the full data set using GenAlex software.

Variation	DF	SS	MS	EV	PV	Stat	Value	P.Val
Among Regions	1	110404.30	110404.30	883.67	47.00	PhiRT	0.47	0.001
Among Pops	5	6396.94	1279.39	11.55	1.00	PhiPR	0.01	0.086
Within Pops	264	260627.30	987.22	987.22	52.00	PhiPT	0.52	0.001
Total	270	377428.50	1397.88	1882.45	100.00			

Key: DF: degree of freedom, SS: Sum of squares, MS: Mean of squares, EV: estimate of variation, PV: percentage variance

### Genetic differentiation and genetic distance

In this study (Table 4), we found high (0.47) and highly significant genetic differentiation (PhiRT) values among the regions. High genetic difference values were found between Vanuatu and Enugu (0.462), Vanuatu and Ebonyi (0.352), Vanuatu and Imo (0.433), Vanuatu and Anambara (0.457), Vanuatu and Abia (0.438), and Vanuatu and NRCRI (0.441). Ebonyi and Enugu (0.107), Ebonyi and Anambara (0.094), Ebonyi and Imo (0.094), Ebonyi and Abia (0.066), and Ebonyi and Imo (0.066) had moderate genetic divergence levels between their taro populations. The remaining population has low and non-significant genetic differentiation. Pairwise Nei’s [34] minimum genetic distance also showed similar pattern among the studied regions (Table 4). The mean genetic distance between Vanuatu and Anambara, Vanuatu and Enugu, Vanuatu and Abia, Vanuatu and Imo, Vanuatu and NRCRI, and Vanuatu and Ebonyi taro populations was 0.22, 0.22, 0.21, 0.20, 0.18, and 0.16, indicating high genetic variation between Vanuatu and Nigerian taro accessions. A moderate genetic distance (0.05) was observed between taro population originating in Ebonyi and Anambara, as well as Ebonyi and Enugu, indicating the presence of genetic variation among the populations. Maximum genetic distances (0.49) were observed between accessions NCe005-8 (originating from NRCRI) and SM120-43 (originating from Vanuatu), while a lower genetic distance (0.10) was observed between EBNFC032 (originating from Anambara) and NCe010-18 (originating from NRCRI) (S3 Table).

**Table 4:** Pairwise PhiPT values (above diagonal) and Nei’s minimum genetic distance (below diagonal) between subgroups among populations within region for 271 taro accessions assessed using GenAlex software.

Region	Enugu	Ebonyi	Imo	Anambara	Abia	NRCRI	Vanuatu
Enugu		0.11**	0.00	0.00	0.00	0.01	0.46**
Ebonyi	0.05*		0.05*	0.09**	0.07*	0.05*	0.35**
Imo	0.02	0.03		0.01	0.00	0.01	0.43**
Anambara	0.02	0.05*	0.02		0.00	0.01	0.46**
Abia	0.02	0.04	0.02	0.02		0.00	0.44**
NRCRI	0.02	0.03	0.02	0.02	0.02		0.44**
Vanuatu	0.22**	0.16**	0.20**	0.22**	0.21**	0.18**	

Key:

*: significant (P<0.05),

**: highly significant (P<0.01)

### Principal coordinate analyysis (PCoA)

**Fig 1:**
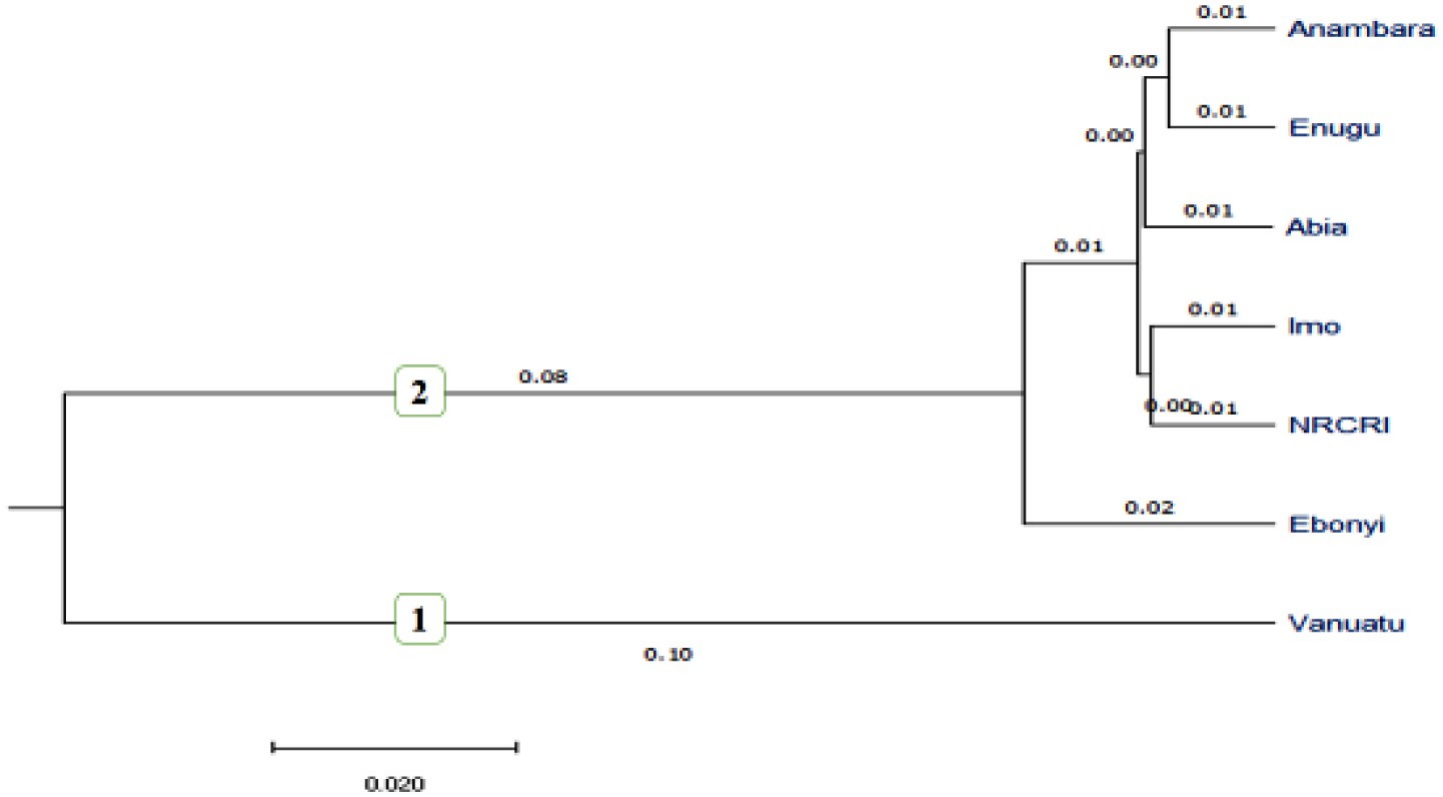
Dendrogram constructed by unweighted pair group method with arithmetic mean (UPGMA) based on region of origins using Euclidian distance (cut off 0.05).

**Fig 2:**
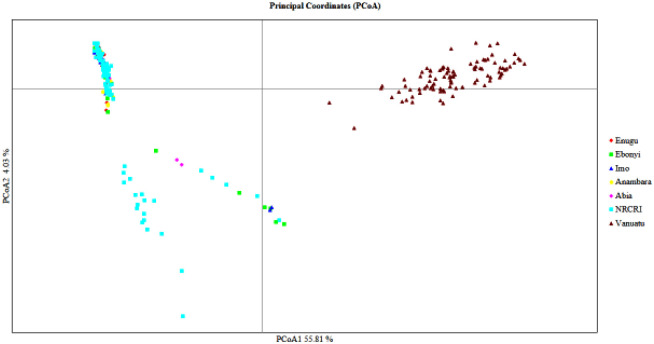
A bi-plot of the first two principal components (PC1 and PC2) of 271 taro accessions, using 10,391 SNP markers, each color corresponds to population structuring and grouping by geographical position.

Principal component and cluster analysis were performed on seven geographical origins, and clustering analysis grouped all geographical origins into two (Fig 1). Cluster 1 included the Vanuatu geographical origin, whereas Cluster 2 included all Nigerian geographical origins. This finding supports the AMOVA result, which found large and highly significant genetic variation between regions. Among the 271 accessions in our study, principal coordinate analysis (PCoA) also revealed the existence of two subgroups (Fig 2).

**Fig 3:**
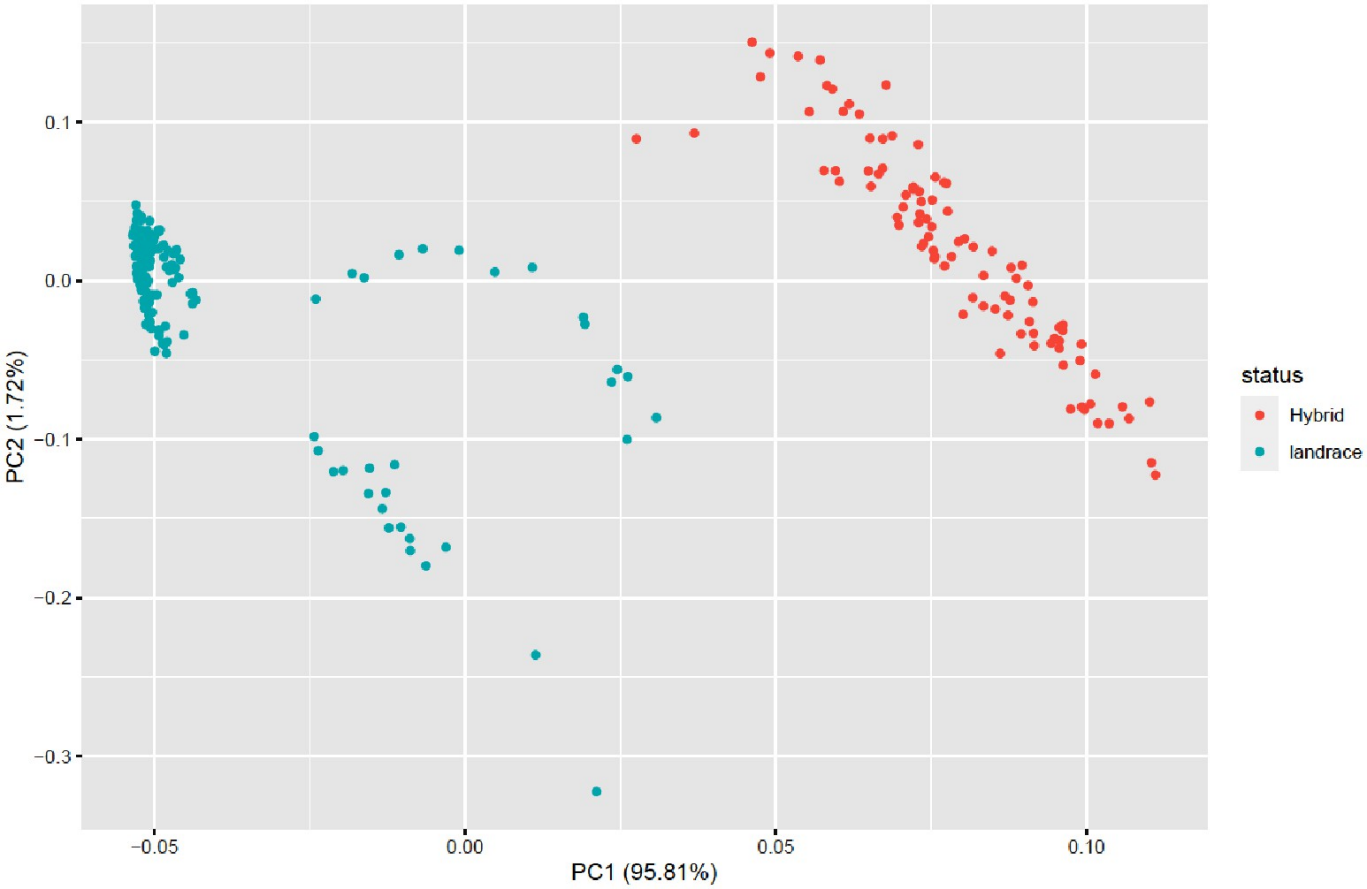
A bi-plot of the first two principal components (PC1 and PC2) of 271 taro accessions, using 10,391 SNP markers, each colour corresponds to population structuring and grouping based on types of population.

All taro accessions were divided into two groups based on their population type (landrace and hybrid) using a 0.05 cut-off Euclidean distance (S1 Fig). Cluster 1 contained 177 (65.31%) taro accessions (landraces) all originating in Nigeria, whereas Cluster 2 (34.68%) contained all taro accessions (hybrids) originating in Vanuatu. Similarly, based on population status (hybrid and landrace) (Fig 3), the first two PCAs explained 97.53% of the variation, with PCA 1 alone accounting for 95.81% of the variation.

### Admixture and Discriminate analysis of principal component (DAPC)

**Fig 4:**
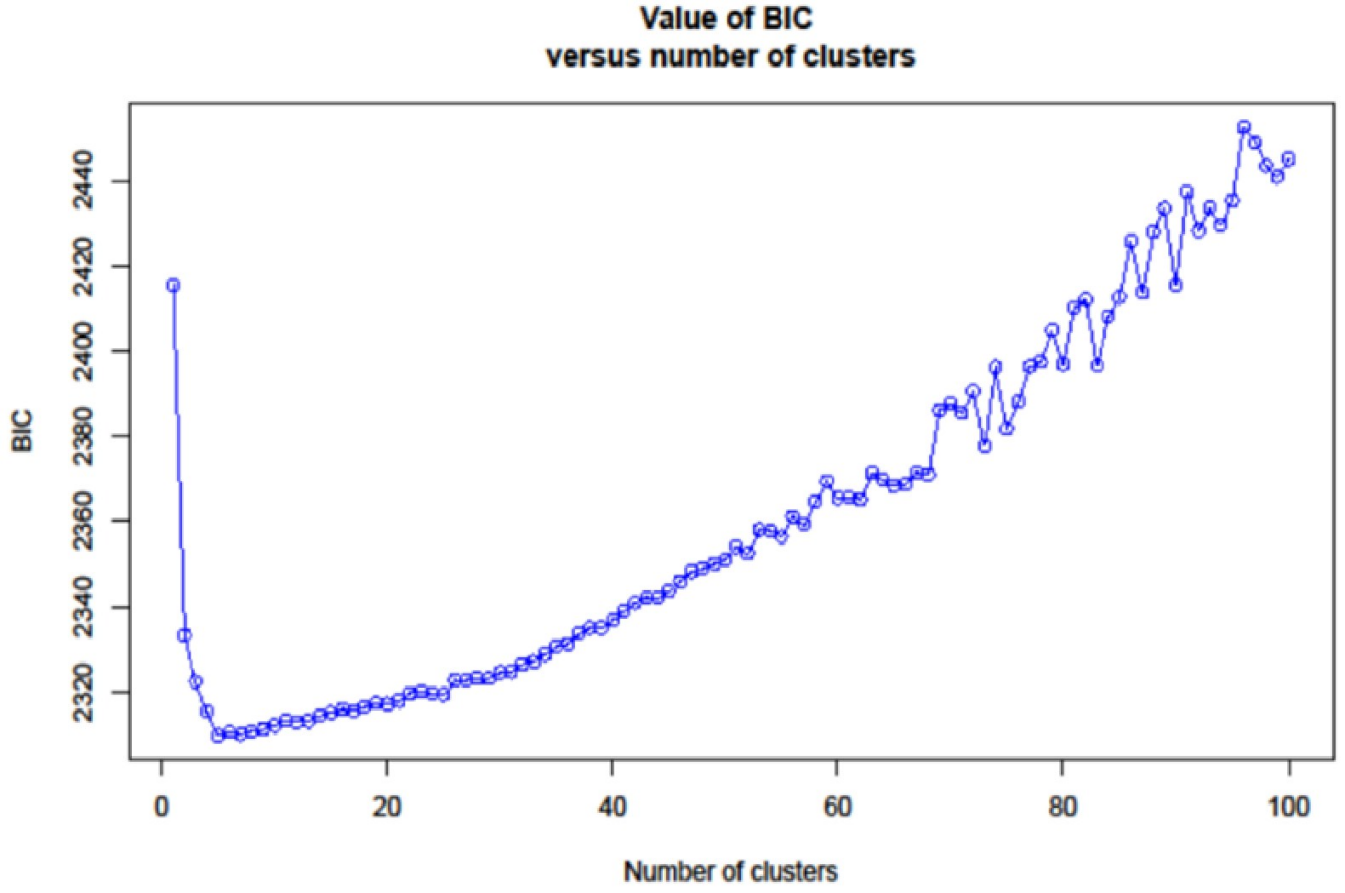
Values of BIC verses number of clusters.

**Fig 5:**
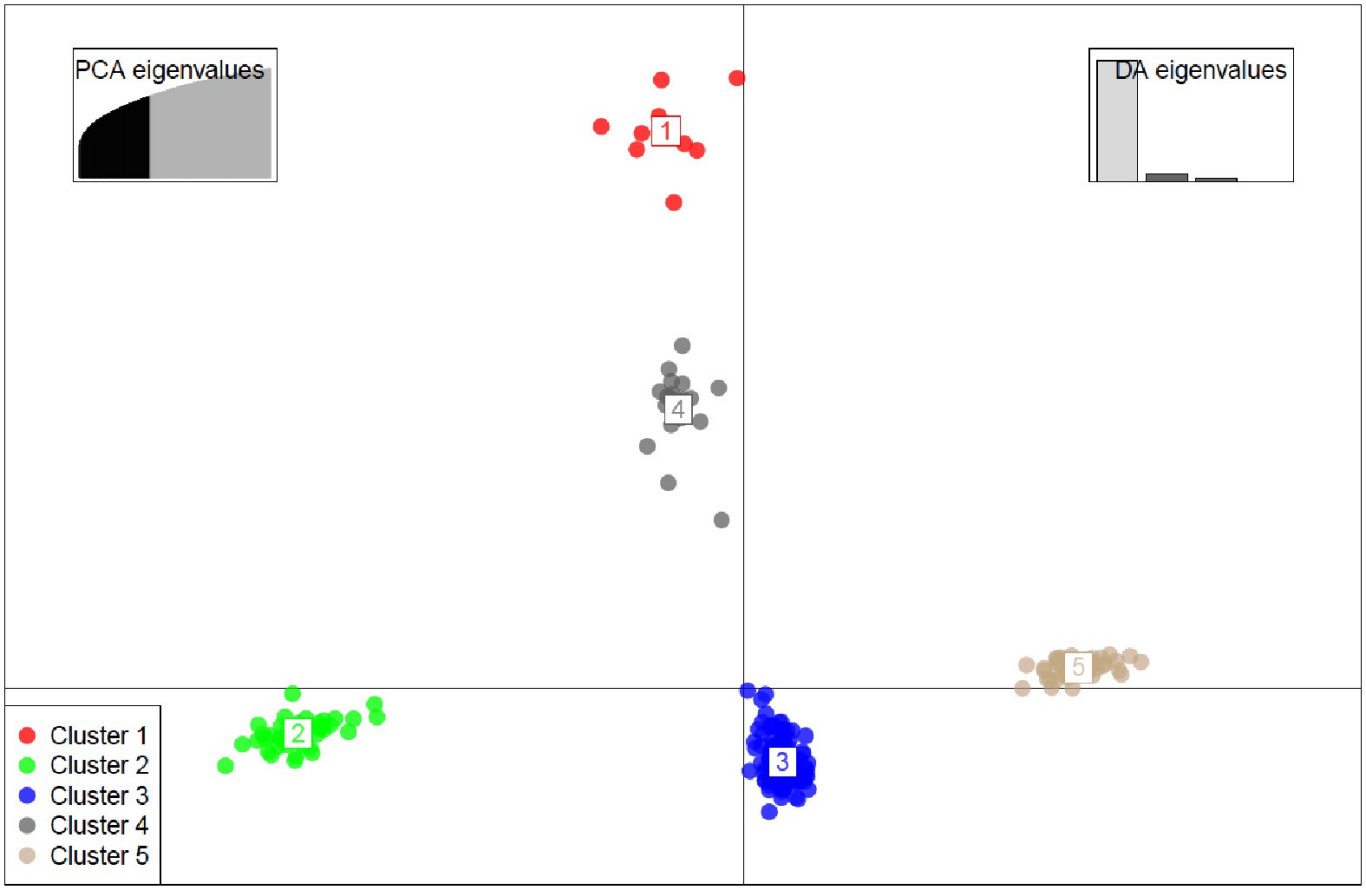
Discriminate analysis of principal component (DAPC) with K = 5.

**Fig 6:**
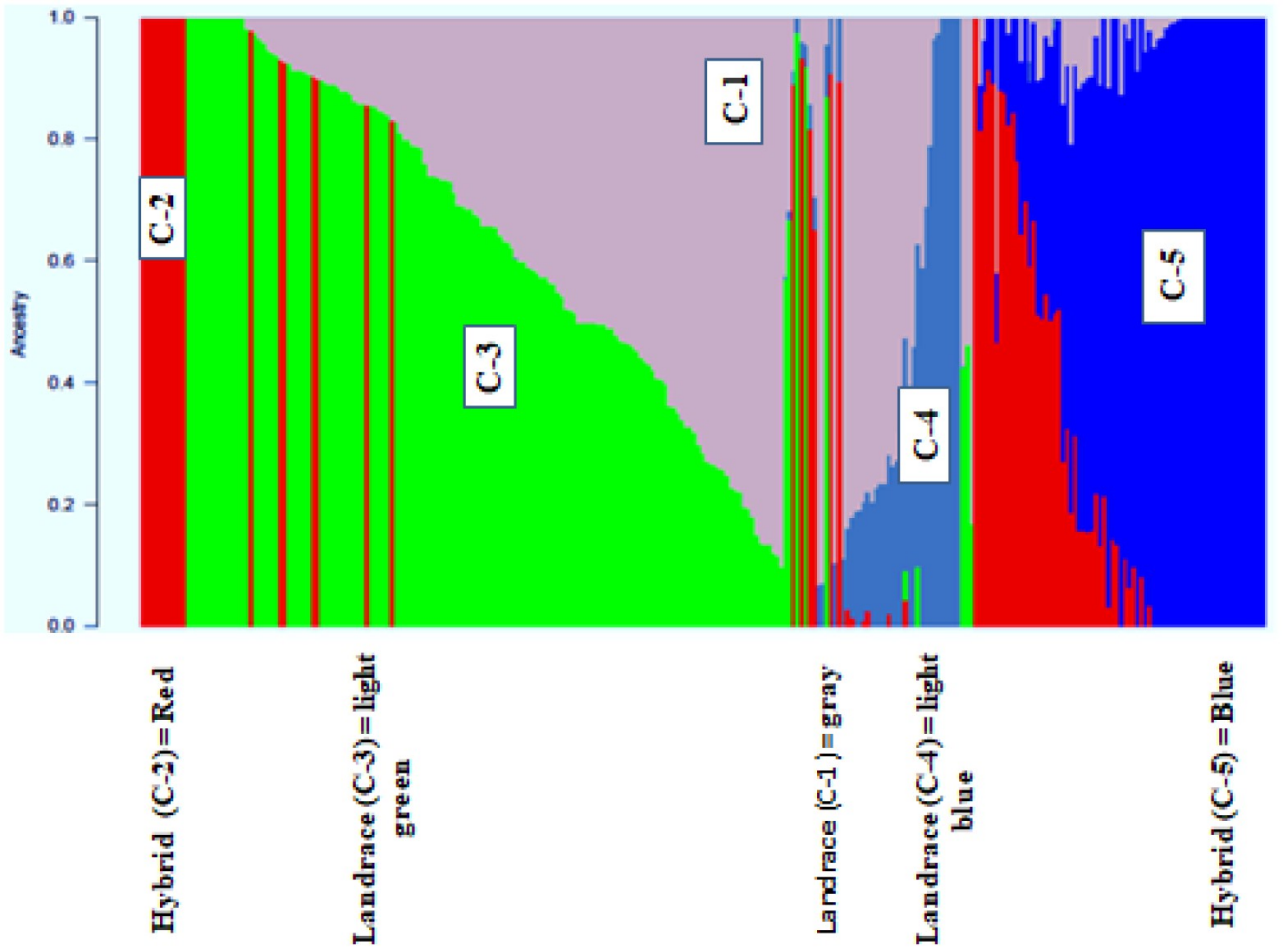
Population structure of 271 taro accessions, K = 5; each colour represents one cluster (C: 1–5).

With a set of 10,391 SNP markers, population structure analysis among 271 taro accessions revealed an optimal K value of five (Fig 4), dividing the diverse panel group into five major clusters (Fig 5 and S4 Table). Cluster one (C1) had 9 accessions, cluster two (C2) had 37, cluster three (C3) had 77, cluster four (C4) had 49, and cluster five (C5) had 47. 80.81% of the accessions were assigned to one of the five subpopulations with an ancestry membership coefficient greater than 0.60 (Fig 6). The remaining 19.19% of accessions (with an ancestry membership coefficient less than 0.6) were identified as admixture accessions, indicating that these populations are evolving and less differentiated. Taro accessions in clusters 1, 3, and 4 are landraces from Nigeria, whereas populations in clusters 2 and 5 are hybrids from Vanuatu. 42 of the 52 admixed taro accessions were sourced from Nigeria, with the remaining ten taro accessions sourced from Vanuatu (S5 Table).

### Relationships between clusters

**Fig 7:**
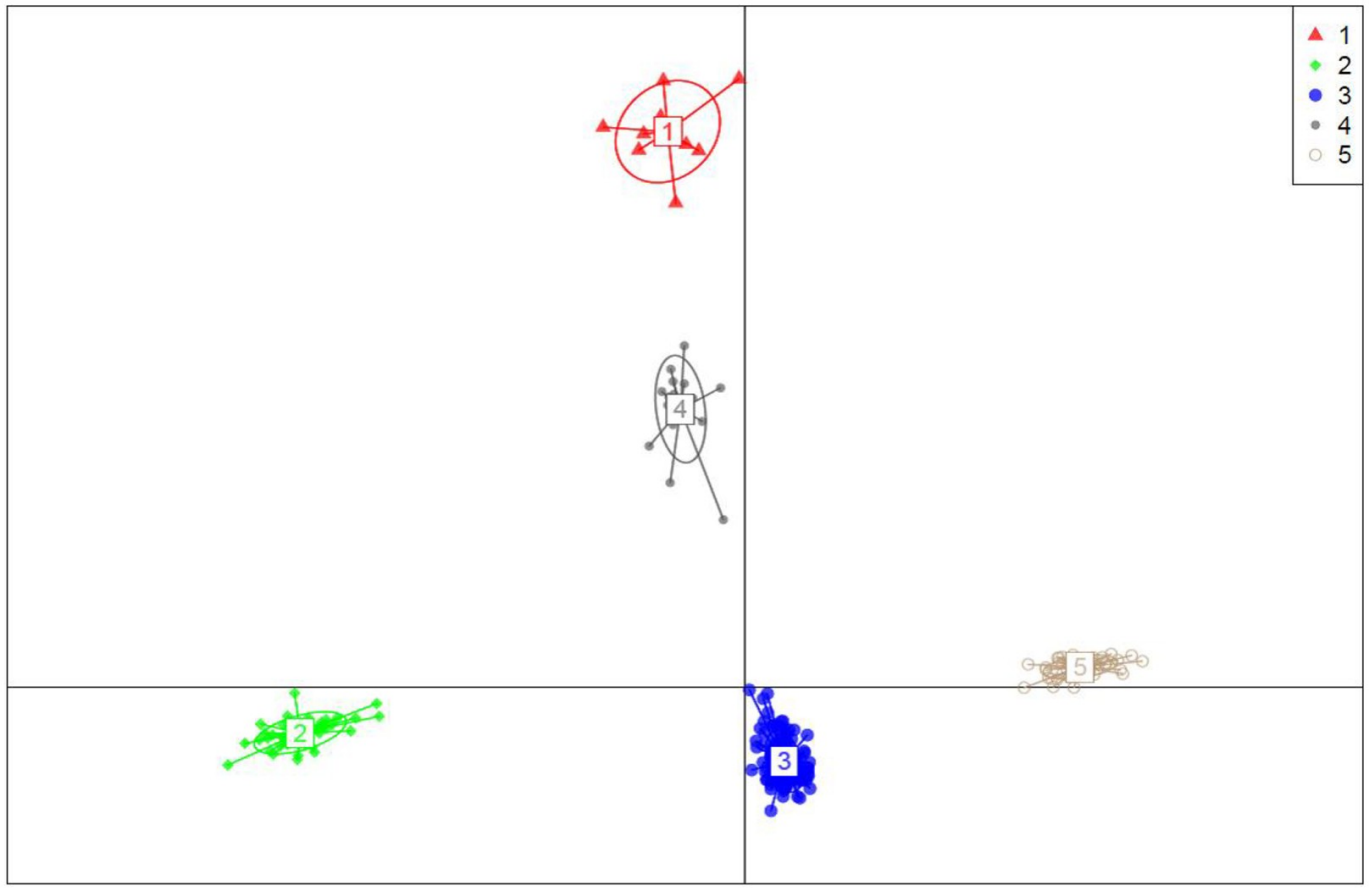
The genetic networks for all genetic groups, with node sizes indicating genetic relationships between different accessions.

The analysis revealed five distinct clusters, three of which are all Nigerian landraces (cluster 1, 3, and 4) (Fig 7). Cluster 2 and 5 represent Vanuatu hybrid accessions. The size of the nods in the accessions represents their cluster relationships. The smaller the node size, the greater the similarity of accessions in the cluster, and vice versa. These findings highlighted the genetic relationships between different genetic groups of taro in Nigeria as well as Vanuatu materials.

## Discussion

### Genotyping

The best way to achieve efficient management of crop genetic resources to improve breeding programs and understand the ancestry relationships of accessions is to reveal the population structure and diversity of a collection [35]. The current study used DArT sequence to examine the diversity and population structure of the Nigerian and Vanuatu taro panels, which included 271 accessions from seven different geographical origins.

In the current study, a total of 32,227 SNPs were initially called from the accessions using a variant calling pipeline. The observed number of SNPs in the current study was high when compared to the report by Soulard et al. [36] but low when compared to the report by Liu et al. [37]. The taro panel studied in this study was characterized using 10,391 high-quality SNPs. In line with this finding, Liu et al. [37] used a large number of SNPs (17,047) to characterize taro genotypes, whereas Soulard et al. [36] and Helmkampf et al. [38] used only 459 and 2400, respectively.

### Allelic proportion

In the current study, the SNP with the most allelic sites and proportion was GA (3102, 29.85%), while the nucleotide CG (907, 8.73%) had the smallest read and proportion (Table 1). Mace and Godwin [39], on the other hand, reported a high GT/CA (42%) repeat motif using microsatellite. Furthermore, we discovered that the proportion of SNP transitions was higher (6,122 allelic sites, 58.92%) than SNP transversions (4269 allelic sites, 41.08%). In true SNPs, transition is more common than tarnsversion, and there may be two transition SNPs out of three available SNPs [40].

The observed and expected heterozygozity in the current study ranged from 0.40 to 0.53 and 0.26 to 0.39, respectively, indicating moderate genetic variability among the taro accessions studied. Similarly, Hu et al. [41] found high observed and expected heterozygozity ranging from 0 to 0.73 and 0.38 to 0.73, respectively, among taro accessions using microsatellite markers. Heterozygozity was higher for Vanuatu taro accessions than Nigerian taro accessions (Table 2), indicating the presence of greater genetic variability among Vanuatu than Nigerian taro accessions. The high heterozygozity and genetic variability in the Vanuatu taro accessions could be a result of their hybrid nature. In the current study, we also found that mean observed heterozygozity (0.47) was higher than expected heterozygozity (0.33), indicating that genotypes have an isolate breaking effect [38]. Based on regional group, Vanuatu groups had a higher MAF (0.35) than Nigerian groups (0.23), indicating that Vanuatu accessions contain more useful genes. Similarly, moderate polymorphism (PIC) was observed for accessions originating from Vanuatu (0.30) while low for Nigeria group (0.20). In line with his findings, Palapala and Akwee [17] reported polymorphic information content values ranging from 0.19 to 0.57 in 25 Kenyan taro genotypes, with an average of 0.41 using SSR markers.

### Genetic diversity and population structure

In this study, we found a high percentage of genetic variation across geographical regions (47%), indicating that regional isolation can be a source of genetic diversity. In marginal populations, genetic differentiation is significantly higher than in the center of the range due to spatial segregation and restricted gene flow [42]. In this regard because the regions of Nigeria and Vanuatu are so far apart, it is possible that limited gene flow is to blame for the region’s high genetic variation. Eckert et al. [43] published similar findings, demonstrating genetic variation as a result of geographic isolation. The low Nei genetic distance observed between the sub groups except Vanuatu might suggest the possible presence of redundant accessions. We also found very high (52%) and highly significant individual genetic variation within the taro panel population. Clonal propagation, which leads to mutation, is one of the most likely causes of variation in the currently assessed taro population [2]. In addition, Vanuatu hybrids emanating from sexual reproduction could have a significant influence on the variation observed. The percentage of molecular variation among individuals within a population (52%) in the current study was low when compared to the genetic variation reported (79%) among East African taro collections assessed using SSR markers [44]. Mezhii et al. [45] also reported 100% genetic variation among individuals within a population for 50 taro accessions collected from India, indicating that high within-population variation is a feature of taro plants. Many researchers have reported significant genetic variation among taro accessions using microsatellite markers [2, 16, 22, 46], RAPD markers [14, 47], AFLP markers [48–51] and Isozymes [52, 53]. Quero et al. [48] on the other hand, reported a narrow genetic base among 450 Vanuatu taro accessions studied with AFLP markers. However, very few studies have been conducted to identify genetic variation among taro accessions using SNP markers [10, 36, 38]. Taro is genetically diverse [54]. The cluster dendrogram UPGMA based on the geographical distribution of accessions in the current study clustered the accessions into two groups, with most taro accessions from the same origin correctly classified on the basis of geographical regions of origin. The current AMOVA analysis also supported the idea that gene flow between regions is less likely because genetic variation among geographical groups accounted for 47% of total variation. According to this history, taro propagate movement was restricted between Nigeria and Vanuatu. In admixture, very few accessions (19.19%) were grouped out of 271 genotyped accessions. Similar results were reported by many researchers on taro. Mezhii et al. [45] for example, reported four distinct clusters with a level of 35% similarity among the individuals.

## Conclusion

This study demonstrated the utility of SNPs in characterizing the genetic diversity and population structure of taro collections. Based on the gene diversity values calculated from the 10,931 SNPs, the Nigerian and Vanuatu accessions appeared to be genetically diverse. Since within-population variation was significant than between populations, we suggest that, during collecting missions, germplasm collectors should increase sampling of more accessions within a location than increasing the number of locations. The degree of genetic relationship and differentiation among genetic resources can be used to increase genetic diversity and combine alleles for valuable agricultural traits. Thus, the collections contain valuable genetic information for future conservation and breeding studies.

## Supporting information

S1 Table List of accessions used in the study. (CSV)

S2 Table SNP marker summary statistics across fourteen chromosomes. (DOCX)

S3 Table Nie genetic distance for 271 taro accessions using 10,391 SNP markers. (CSV)

S4 Table Clusters and adimixture of 271 taro accessions studied. (CSV)

S5 Table Proportion of admixture by regions, proportion and types of population. (DOCX)

S1 Fig NJ tree of 271 taro accessions, using 10,391 SNP markers. (DOCX)

S1 Data (CSV)
