# Supplementary material for: DArTSeq SNP-based genetic diversity and population structure studies among taro [(Colocasia esculenta (L.) Schott] accessions sourced from Nigeria and Vanuatu
Source: PLoS One. 2022 Nov 10;17(11):e0269302. doi: 10.1371/journal.pone.0269302 (PMC9648780; doi:10.1371/journal.pone.0269302)
Supplement: S2 Table — (DOCX) [file pone.0269302.s002.docx]

**S2 Table. SNP marker summary statistics across fourteen chromosomes**

| **Chromosome** | **No of SNPs** | **PIC** | **MAF** | **Ho** | **He** |
| --- | --- | --- | --- | --- | --- |
| 1 | 8811 | 0.27 | 0.27 | 0.45 | 0.34 |
| 2 | 749 | 0.27 | 0.27 | 0.44 | 0.34 |
| 3 | 320 | 0.27 | 0.27 | 0.45 | 0.34 |
| 4 | 162 | 0.27 | 0.27 | 0.46 | 0.34 |
| 5 | 106 | 0.27 | 0.29 | 0.48 | 0.35 |
| 6 | 75 | 0.28 | 0.31 | 0.54 | 0.36 |
| 7 | 42 | 0.26 | 0.25 | 0.40 | 0.32 |
| 8 | 25 | 0.26 | 0.30 | 0.52 | 0.33 |
| 9 | 22 | 0.25 | 0.24 | 0.40 | 0.32 |
| 10 | 44 | 0.28 | 0.28 | 0.47 | 0.35 |
| 11 | 21 | 0.26 | 0.25 | 0.39 | 0.32 |
| 12 | 10 | 0.32 | 0.37 | 0.65 | 0.42 |
| 13 | 1 | 0.37 | 0.46 | 0.83 | 0.50 |
| 14 | 3 | 0.29 | 0.35 | 0.57 | 0.38 |
| **Grand Total** | **10391** | **0.27** | **0.27** | **0.45** | **0.34** |

PIC: polymorphic information content; MAF: minor allele frequency; Ho: observed heterozygosity; He: expected heterozygosity
