# Supplementary material for: DArTSeq SNP-based genetic diversity and population structure studies among taro [(Colocasia esculenta (L.) Schott] accessions sourced from Nigeria and Vanuatu
Source: PLoS One. 2022 Nov 10;17(11):e0269302. doi: 10.1371/journal.pone.0269302 (PMC9648780; doi:10.1371/journal.pone.0269302)
Supplement: S5 Table — (DOCX) [file pone.0269302.s005.docx]

**S5 Table. Proportion of admixture by regions, proportion and types of population**

| **Regions** | **Number of accessions** | **Admixed individuals** | **Proportion (%)** | **Types of population** |
| --- | --- | --- | --- | --- |
| Ebonyi | 15 | 3 | 20.00 | Landrace |
| Anambara | 20 | 5 | 25.00 | Landrace |
| Enugu | 20 | 4 | 20.00 | Landrace |
| Abia | 17 | 3 | 17.65 | Landrace |
| Imo | 20 | 5 | 25.00 | Landrace |
| NRCRI | 85 | 22 | 25.88 | Landrace |
| Vanuatu | 94 | 10 | 10.64 | Hybrid |
| **Total** | **271** | **52** | **19.19** |  |
