# Supplementary figures and images for: DArTSeq SNP-based genetic diversity and population structure studies among taro [(Colocasia esculenta (L.) Schott] accessions sourced from Nigeria and Vanuatu
Source: PLoS One. 2022 Nov 10;17(11):e0269302. doi: 10.1371/journal.pone.0269302 (PMC9648780; doi:10.1371/journal.pone.0269302)

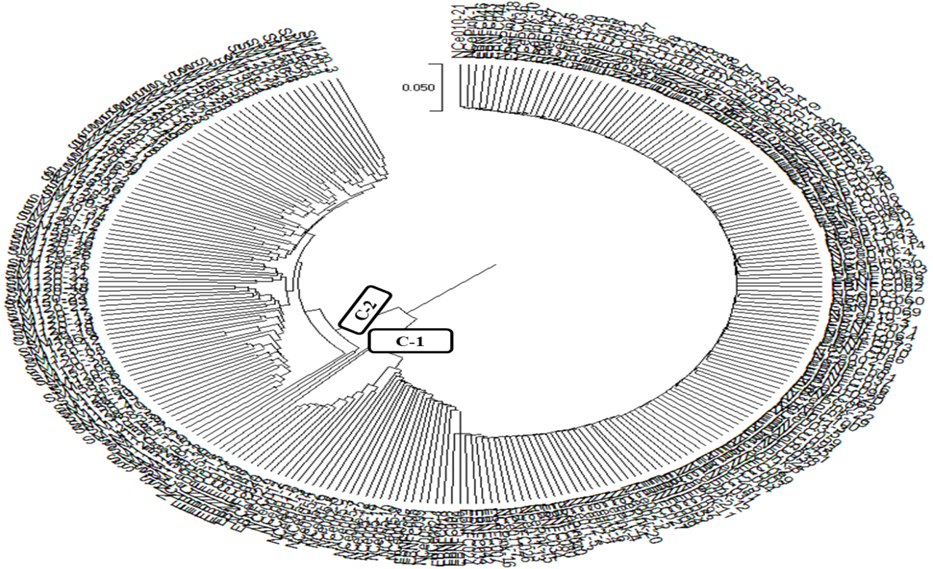


S1 Fig. NJ tree of 271 taro accessions, using 10,391 SNP markers

Supplement: S1 Fig — (DOCX) [file pone.0269302.s006.docx]
